# Perinatal Stroke Presenting as Arm Swelling: A Case Report

**DOI:** 10.5811/cpcem.21246

**Published:** 2025-02-15

**Authors:** Sarah K. Sylvester, Amber M. Morse, Ryan C. Kwong

**Affiliations:** University of Arkansas for Medical Sciences, Department of Pediatrics, Section of Pediatric Emergency Medicine, Little Rock, Arkansas

**Keywords:** perinatal stroke, pediatric, ultrasound, magnetic resonance imaging, case report

## Abstract

**Introduction:**

Perinatal stroke is a rare but clinically significant condition that can present in a variety of ways and can result in diagnostic challenges in a particularly vulnerable population. We present the case of a term neonate who presented with left arm swelling, ultimately diagnosed with perinatal stroke.

**Case Report:**

A term male neonate presented to the emergency department with left arm swelling noted the day prior, with abnormal tone of the left arm since birth. Physical examination revealed mild erythema and edema localized to the left upper extremity, with the arm held in flexion. Neurological examination was otherwise unremarkable. Further evaluation, including imaging studies, demonstrated thrombi in the left axillary and subclavian arteries, as well as an infarct involving the right middle cerebral artery and anterior cerebral artery with diffusion restriction, consistent with perinatal stroke.

**Conclusion:**

Through this case report, we aimed to increase awareness of perinatal stroke among healthcare professionals and highlight the importance of prompt recognition and appropriate management in optimizing outcomes for affected infants.

## INTRODUCTION

Perinatal stroke, which occurs between 20 weeks gestation and 28 days of life, is a neurological emergency associated with significant morbidity and mortality. Although the exact incidence remains uncertain, perinatal stroke is estimated to occur in 1 in 1600–2300 live births.^1^ Approximately half of perinatal strokes present acutely in the first days of life.^1^,^2^ Perinatal stroke poses substantial challenges in diagnosis and management due to its diverse clinical presentations and limited evidence-based guidelines. Despite advances in medical imaging and diagnostic techniques, the diagnosis of perinatal stroke can be difficult, often leading to delays in recognition and management. In this case report, we discuss the presentation of a patient with perinatal stroke, highlighting the complexities in diagnosis and management.

## CASE REPORT

A six-day-old term male presented to our emergency department (ED) with left arm swelling. He was born via spontaneous vaginal delivery to a 17-year-old, gravida one para one, at 38 weeks two days gestational age, weighing 2.466 kg. It was noted that he had acrocyanosis of the left arm at birth, resolved after several minutes on the warmer. Apgar scores were 8 and 9 at one and five minutes of life, respectively, out of a maximum score of 10. Nursing staff additionally noted that his arm had no spontaneous movement immediately after birth with decreased tone, and he was subsequently diagnosed with a presumed nerve palsy. Mother reported he had been “stuck in the birth canal” during delivery. Chest radiograph obtained while in the nursery was negative for fracture. Prior to discharge from the nursery at day two of life, he was noted to have improvement in his movement of the left arm.

He was seen by his primary care physician (PCP) at four days of age, where he was noted to have improved movement of the left arm. At five days of age, his mother noted that his left arm appeared swollen. He was seen at an outside ED and diagnosed with ulnar nerve palsy. Following a routine visit to his PCP for bilirubin check on the day of presentation, the family was encouraged to present to our ED for further evaluation of arm swelling.

**Image 1:**
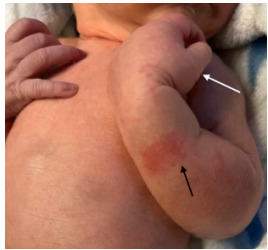
Patient’s left arm was held in a flexed position with an area of forearm erythema (black arrow) and distal extremity edema (white arrow).

On presentation to our ED, examination was remarkable for the left arm held in flexion at the elbow with edema to the forearm and hand (Image 1), but no appreciable tenderness to palpation. Capillary refill was brisk, and radial pulses were 2+ and symmetric. He was noted to have erythema to the left forearm that had been present since birth. Radiographs of the right humerus and forearm were unremarkable. Ultrasound of the left upper extremity was obtained due to concern for thrombosis and revealed a long non-occlusive thrombus within the left axillary artery measuring approximately one centimeter in length, as well as a few small, non-occlusive adherent thrombi anteriorly and posteriorly in the midportion of the subclavian artery, with a distal area of turbulent arterial flow favored to be a thrombus.

**Image 2:**
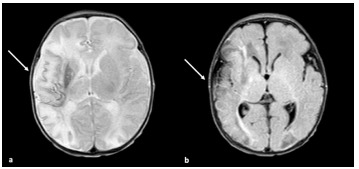
Magnetic resonance imaging T2 (a) and fluid-attenuated inversion recovery (b) demonstrating hyperintensity and diffusion restriction involving the right middle cerebral artery and anterior cerebral artery distribution consistent with infarct (white arrows).

Hematology was consulted and they made imaging recommendations. A magnetic resonance imaging (MRI) of the brain demonstrated a large acute/subacute infarct involving the right middle cerebral artery (MCA) and anterior cerebral artery with significant diffusion reduction. A magnetic resonance angiogram of the brain demonstrated severe focal occlusion at the M1/M2 junction of the MCA with normal flow seen in the distal MCA branches. Magnetic resonance venography of the brain was normal (Image 2). Laboratory studies were remarkable for significant elevation in D-dimer of 1,248 nanograms per milliliter (ng/mL) (reference range: < 500 ng/mL), low fibrinogen level of 148 milligrams per deciliter (mg/dL) (200 to 393 mg/dL), and hypoglycemia of 49 mg/dL (70–105 mg/dL). Hypoglycemia was corrected with dextrose-containing fluids. The patient was then admitted to the neonatal intensive care unit (NICU).

**CPC-EM Capsule**

What do we already know about this clinical entity?

*Neonatal stroke is a rare but significant neurological emergency that necessitates timely diagnosis and management*.

What makes this presentation of disease reportable?

*This presentation is unusual as the neonate presented with extremity swelling and was found to have a clot, which led to workup that identified a neonatal stroke*.

What is the major learning point?

*A high index of suspicion, comprehensive diagnostic evaluation, and multidisciplinary management is needed to optimize outcomes*.

How might this improve emergency medicine practice?

*Increased recognition of neonatal stroke among emergency physicians is essential for early intervention to improve the long-term prognosis of affected infants*.

Neonatology, neurology, and hematology came to a consensus on day one of hospitalization that heparin therapy should not be initiated as it was felt that any potential benefit of anticoagulation did not outweigh the risk of hemorrhagic transformation, with further increased risk due to the large infarct. On hospital day three, repeat ultrasound of the left upper extremity remained unchanged from the prior study. Head ultrasound showed increased echogenicity in the right ACA and MCA consistent with known infarcts without hemorrhage. Echocardiogram demonstrated a structurally normal heart, a patent foramen ovale with a small left to right shunt, and no intracardiac thrombus.

Interventional radiology was consulted on hospital day five and did not recommend angiography or arterial intervention as the arterial thrombus was small and non-occlusive on ultrasound, and he maintained good perfusion to the extremity on examination. Additional laboratory studies were obtained, including protein S activity, which was elevated at 70% and antithrombin activity, which was low at 48%, although noted to be expected in neonates. Protein C activity and protein S antigen were unable to be obtained and testing was deferred to hematology on an outpatient basis at a later date.

While in the NICU, the patient underwent physical therapy and had improvement in movement of the left arm with the exception of the hand, which he was unable to extend fully although finger movement was noted. Orthopedic surgery was also consulted and recommended outpatient follow-up in the brachial plexus clinic. He was discharged from the NICU on hospital day eight with outpatient follow-up scheduled with hematology, orthopedics, and neurology.

## DISCUSSION

The diagnosis of perinatal stroke poses several challenges due to its non-specific clinical presentation and the limitations of neuroimaging modalities in neonates. Common presenting symptoms in neonates can include seizures, recurrent apnea or desaturations, alteration in tone, altered level of consciousness, and focal neurological deficits.^2^ Cranial ultrasound is often the initial imaging modality used in perinatal stroke, but its sensitivity for detecting ischemic lesions is limited, especially in the early stages.^2^,^3^ Magnetic resonance imaging with diffusion-weighted imaging is considered the gold standard for diagnosing perinatal stroke, offering superior sensitivity and specificity compared to other imaging modalities including computed tomography (CT). However, if MRI is not readily available or impractical, CT or ultrasound should be used.^3^

The management of perinatal stroke focuses on supportive care, seizure control, and prevention of secondary complications. Anticoagulation therapy, which is commonly used in adult stroke management, is controversial in neonates due to the risk of intracranial hemorrhage and is not routinely indicated.^2^ Instead, management strategies often include hydration, temperature regulation, and physical and occupational therapy to optimize neurodevelopmental outcomes.^2^,^3^

Long-term outcomes following perinatal stroke vary widely, ranging from complete recovery to severe neurodevelopmental disabilities.^2^,^4^ Early identification of neurodevelopmental delays and prompt initiation of early intervention services are crucial in optimizing outcomes for affected infants.^1^,^4^ Multidisciplinary collaboration and follow-up involving neurology, hematology, and rehabilitation specialists is essential.

## CONCLUSION

Perinatal stroke is a neurological emergency that necessitates timely diagnosis and management. Further study is needed to elucidate the underlying mechanisms, refine diagnostic approaches, and develop targeted therapeutic interventions. Increased awareness among clinicians is essential to facilitate early intervention and improve the long-term prognosis of affected infants. This case underscores the need for a high index of suspicion, comprehensive diagnostic evaluation, and multidisciplinary management to optimize outcomes in affected infants.
